# Testing the Influence of Incomplete DNA Barcode Libraries on Ecological Status Assessment of Mediterranean Transitional Waters

**DOI:** 10.3390/biology10111092

**Published:** 2021-10-25

**Authors:** Maurizio Pinna, Benedetta Saccomanno, Gabriele Marini, Francesco Zangaro, Akbota Kabayeva, Mina Khalaj, Laura Shaimardan, Simona D’Attis, Eftychia Tzafesta, Valeria Specchia

**Affiliations:** 1 Department of Biological and Environmental Sciences and Technologies, University of Salento, DiSTeBA, Via Monteroni 165, 73100 Lecce, Italy; maurizio.pinna@unisalento.it (M.P.); benedetta.saccomanno@unisalento.it (B.S.); gabriele.marini@unisalento.it (G.M.); francescozangaro1@gmail.com (F.Z.); simonadattis@libero.it (S.D.); eftychia.tzafesta@unisalento.it (E.T.); 2 Research Centre for Fisheries and Aquaculture of Aquatina di Frigole, DiSTeBA, University of Salento, 73100 Lecce, Italy; 3 Department of Biotechnology, Al-Farabi Kazakh National University, Al-Farabi Avenue, Almaty 71-050040, Kazakhstan; akbota.akhmetkali@gmail.com (A.K.); laush.94@gmail.com (L.S.); 4 Department of Basic Sciences, Sari Agricultural Sciences and Natural Resources University, 48 Sari, Iran; mina.khalaj1989@gmail.com

**Keywords:** benthic macroinvertebrates, DNA-based methods, incomplete DNA barcode reference libraries, Mediterranean transitional waters, ecological status assessment, biomonitoring programs

## Abstract

**Simple Summary:**

The biodiversity and ecological status assessment of transitional water ecosystems by benthic macroinvertebrates investigation could use DNA barcode tools for more rapid and efficient outputs. The principal limits of this application are the incompleteness of DNA barcode databases, the identification of optimal primers set, and the gap in the species sequences. The influence of the incompleteness of DNA barcode libraries on species diversity indices, ecological indicators, and ecological status assessment in transitional waters of the southeast Mediterranean were analysed, underlying the importance to implement DNA barcode libraries and to put an effort toward specific species at a local level.

**Abstract:**

The ecological assessment of European aquatic ecosystems is regulated under the framework directives on strategy for water and marine environments. Benthic macroinvertebrates are the most used biological quality element for ecological assessment of rivers, coastal-marines, and transitional waters. The morphological identification of benthic macroinvertebrates is the current tool for their assessment. Recently, DNA-based tools have been proposed as effective alternatives. The main current limits of DNA-based applications include the incompleteness of species recorded in the DNA barcode reference libraries and the primers bias. Here, we analysed the influence of the incompleteness of DNA barcode databases on species diversity indices, ecological indicators, and ecological assessment in transitional waters of the southeast Mediterranean, taking into account the availability of commonly sequenced and deposited genomic regions for listed species. The ecological quality status assigned through the potential application of both approaches to the analysed transitional water ecosystems was different in 27% of sites. We also analysed the inter-specific genetic distances to evaluate the potential application of the DNA metabarcoding method. Overall, this work highlights the importance to expand the barcode databases and to analyse, at the regional level, the gaps in the DNA barcodes.

## 1. Introduction

A key global challenge in the 21st century is to maintain the supply of clean water and other aquatic ecosystem services without affecting the supporting biodiversity and ecosystem processes that underpin their sustainability. Accordingly, extensive national and international regulations have been adopted to protect aquatic ecosystems and water resources, including the Water Framework Directive (EC, 2000, Directive 2000/60/EC-WFD), the Marine Strategy Framework Directive (EC, 2008, Directive 2008/56/EC-MSFD), the Swiss Water Protection Ordinance (WPO, Swiss Federal Council 1998), the Clean Water Act (CWA, 1972) of the US Environmental Protection Agency, and the United Nations Convention on the Law of the Sea (UNCLOS, 1982). All of these regulations aim to protect aquatic ecosystems and restore altered ecosystems at least to the category of “good status”, which is defined as a condition slightly altered by anthropogenic activities [1]. The WFD indicates specific biological quality elements (BQEs), such as fish, benthic macroinvertebrates, and phytoplanktonic benthos to assess the ecological quality status (EQS). To evaluate these elements, the directive requires the development and validation of tools mainly based on taxonomic identification and composition, abundance, and species sensitivity [2,3,4,5,6,7,8]. They also include descriptors based on the individual body size [9,10,11,12], functional rates, such as the decomposition of dead organic matter [13,14,15,16,17,18,19,20,21], and ecosystem thermodynamics [22].

Among biological quality elements, the benthic macroinvertebrates are effective biological indicators that respond to environmental changes of aquatic ecosystems [23,24]. Their main characteristics as bio-indicators include poor mobility, high number of species and functional groups, long life-cycles, and their important role in aquatic trophic networks [25,26,27,28]. However, the main weaknesses in the use of benthic macroinvertebrates are the time-consuming process of taxonomic identification and possible misidentifications [29,30,31]. In recent years, DNA-based taxon identification has been emerging as a tool that could improve the accuracy and efficiency of biomonitoring, including the assessment of benthic macroinvertebrate communities [32,33,34,35,36,37,38]. The advantages of DNA-based tools include the identification of immature stages that lack recognizable features and the greater resolution at species level, especially for macroinvertebrates that present a high diversity, in comparison with other groups of species.

The DNA-based method uses specific genomic sequences or “barcodes” that uniquely identify the species [39,40,41]. For animals, the most commonly used and reliable barcode regions belong to the mitochondrial *cytochrome c oxidase* subunit I gene (COI) and rDNA genes [1,39,42,43,44]. Public database repositories, such as BOLD Systems and NCBI GenBank, currently hold a wide range of species based on the reference sequence. However, the use of DNA barcoding currently finds its weaknesses in the incompleteness of these databases and in the primers bias [35,45,46]. Concerning the gap in the taxonomic coverage of the DNA-reference libraries, an example is that only about 48% of benthic macroinvertebrate species included in the most recent AZTI’s Marine Biotic Index list (AMBI list) present at least one DNA barcode sequence in the reference libraries. Another example underlining the gap in the taxonomic coverage of the DNA-reference libraries is that, for the marine benthic macroinvertebrates of the AMBI list, the three most represented phyla (Annelida, Mollusca, and Arthropoda, which represent about 85% of the total species considered in the list) are moderately represented in the DNA barcode reference libraries (from 40 to 50%). On the contrary, a few groups constituted by Nemertea, Sipuncula, and Echinodermata are more represented (at least 65%) in the reference libraries [45]. Concerning the primers bias, the application of DNA metabarcoding ideally requires the identification of primer pairs that are useful for the detection of all taxa present in an environmental sample [35].

The aim of this research is to evaluate the current potential applicability of DNA-based tools in the assessment of the ecological status of transitional water ecosystems at a regional scale, taking into account the gaps in the barcode databases and the availability of useful primers sets. We used a checklist of benthic macroinvertebrates collected in two seasons from ten southeast Mediterranean transitional waters (Apulia, Italy) to compare and to correlate species diversity, ecological indicators, and assessment scores calculated for morphologically identified taxa and for taxa available in the reference libraries.

## 2. Materials and Methods

### 2.1. Benthic Macroinvertebrates Database

**Figure 1:**
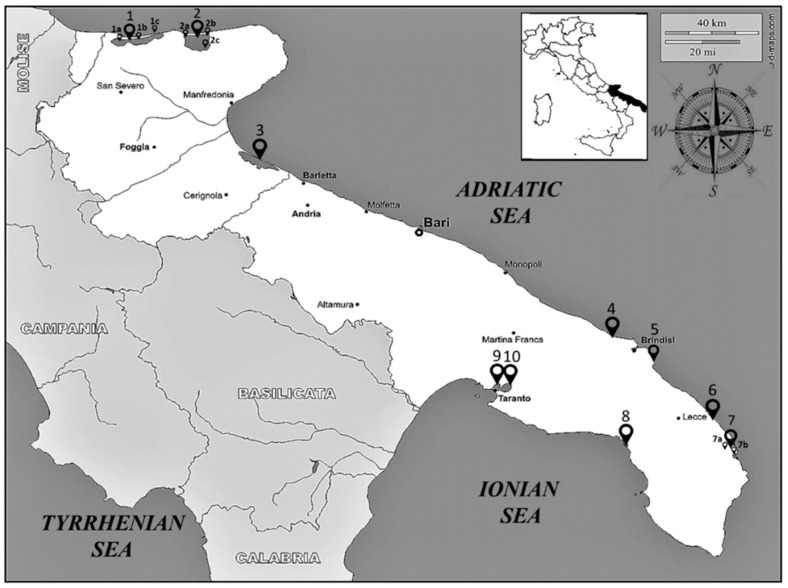
Map of the transitional aquatic ecosystems sampled in the Apulia region (South-East Italy). The numbers on the map correspond to the following ecosystems and sampling sites of each transitional water ecosystem: Laguna di Lesina-1a, 1b, 1c; Lago Varano-2a, 2b, 2c; Vasche Evaporanti, Lago Salpi-3; Torre Guaceto-4; Punta della Contessa-5; Cesine-6; Alimini Grande-7a, 7b; Baia di Porto Cesareo-8; Mar Piccolo-Primo Seno-9; Mar Piccolo-Secondo Seno-10.

For this study, the species checklist of the Apulia Regional Environmental Protection Agency (ARPA-Puglia, Italy) published in 2011 was acquired, consisting of a checklist of benthic macroinvertebrate species from the transitional waters of the Apulia region in the southeast of Italy. An overall number of ten transitional water ecosystems was considered (Figure 1), namely: Laguna di Lesina (1); Lago Varano (2); Vasche Evaporanti, Lago Salpi (3); Torre Guaceto (4); Punta della Contessa (5); Cesine (6); Alimini Grande (7); Baia di Porto Cesareo (8); Mar Piccolo-Primo Seno (9); Mar Piccolo-Secondo Seno (10). A total of fifteen sampling sites were investigated, three in the Laguna di Lesina (1a, 1b, 1c), three in the Lago di Varano (2a, 2b, 2c), two in the Alimini Grande (7a, 7b), and one in each of the other transitional water ecosystems. The data analysed refer to the samplings carried out during the fall of 2010 and the spring of 2011. Each species was defined by its presence or absence in the sampled transitional aquatic ecosystems (Supplementary Table S1). Taxon names of each reported species were verified using both the European platform EU-NOMEN (http://www.eu-nomen.eu) (accessed on 15 March 2021) and the worldwide platform WORMS (http://www.marinespecies.org) (accessed on 15 March 2021). The data matrix was updated to the most recent and accepted taxonomy. DNA barcode libraries (BOLD systems, http://www.boldsystems.org (accessed on 22 March 2021); Ranasignham and Hebert 2007; and NCBI GenBank, https://www.ncbi.nlm.nih.gov/genbank (accessed on 22 March 2021)) were examined in order to produce a reduced database containing all the barcoded species that were needed for our analysis.

### 2.2. Descriptors, Ecological Indicators, and Correlations between Morphological and Reduced Databases

The species richness and the Shannon diversity index were calculated for both databases. Two ecological indicators, AMBI and M-AMBI, were estimated using the AZTI-AMBI software v6.0 (http://ambi.azti.es, access on 24 March 2021), which was used to assess the ecological quality status of transitional water ecosystems by classifying them into five quality categories (High, Good, Moderate, Poor, and Bad). Correlation tests between ecological indicators of morphological and molecular data were carried out to compare the results.

### 2.3. Species Delimitation Analysis

COI sequences of the taxa listed in Table S1 were downloaded from BOLD Systems and NCBI using PrimerMiner 0.3b [47,48,49]. We only omitted taxa where the species was not specified or was not present in the database, giving a total of 38 taxa. For each of them, we aligned the sequences of all the accessions available in the databases to generate one consensus sequence, with the only exceptions of *Nereis falsa, Eunice vittate*, and *Actinia fragacea* where only one accession was found. These consensus sequences were then aligned to construct a maximum likelihood tree (ML) in RAxml (Randomized Axelerated Maximum Likelihood), using the general time reversible + gamma (GTR+G) model [50] and a neighbor-joining (NJ) bootstrap method with the Kimura-2-parameter model in MEGAX. Both trees were then used as constraint trees for species delimitation with bPTP, the Bayesian implementation of the Poisson Tree Processes (PTP) model with a 100,000 MCMC generation and a 1% burn-in [51].

## 3. Results

### 3.1. Presence of Target Species in DNA Barcode Libraries

We examined a total number of 82 benthic macroinvertebrate species within six phyla from ten Apulian transitional water sites (Figure 1 and Table S1) that were reported in the ARPA-Puglia checklist. Among these, only 53 species, corresponding to the 64% of the benthic macroinvertebrates checklist, presented at least one DNA barcode sequence within the BOLD or NCBI GenBank reference libraries. For the remaining 29 species, (36%), a DNA barcode was not available in the reference libraries. The phyla that show a relevant gap in the reference libraries were Mollusca (45%), Annelida (28%), and Arthropoda (21%). These species were also characterized by a reduced occurrence and low density. In particular, the highest number of species without a DNA barcode was found in two of the sampled transitional water ecosystems: Lago di Varano and Mar Piccolo-Secondo Seno.

### 3.2. Species Richness

**Figure 2:**
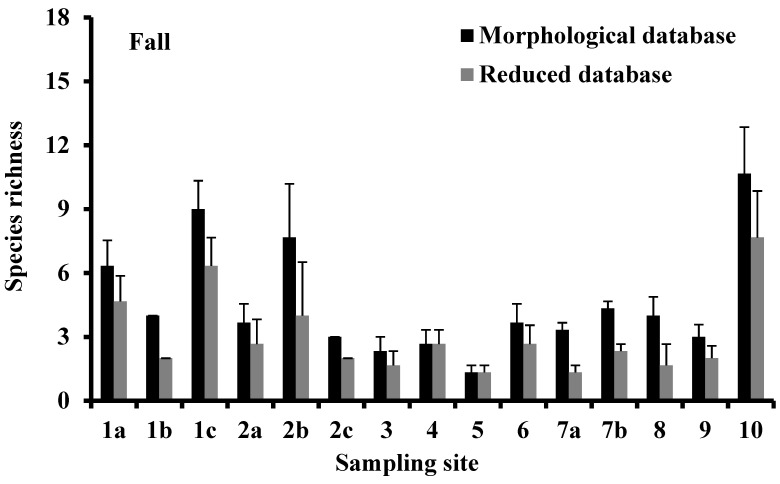

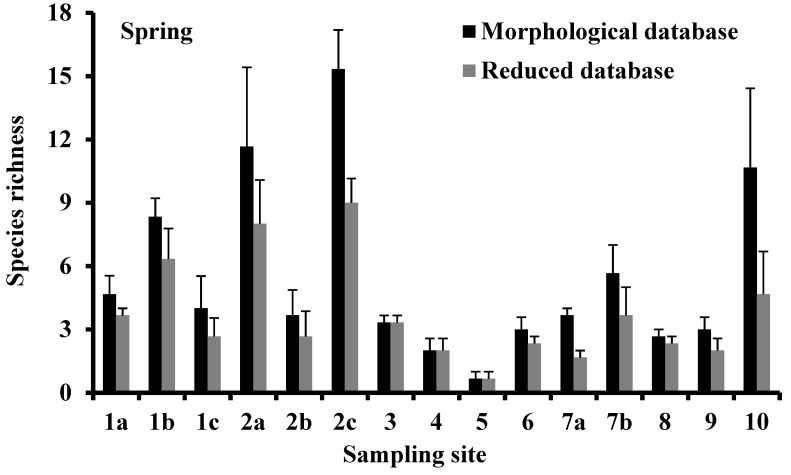
Variation of species richness in the sampling sites of the Apulia transitional waters where the benthic macroinvertebrates were collected during the fall of 2010 and the spring of 2011. Vertical bars indicate the standard error.

**Figure 3:**
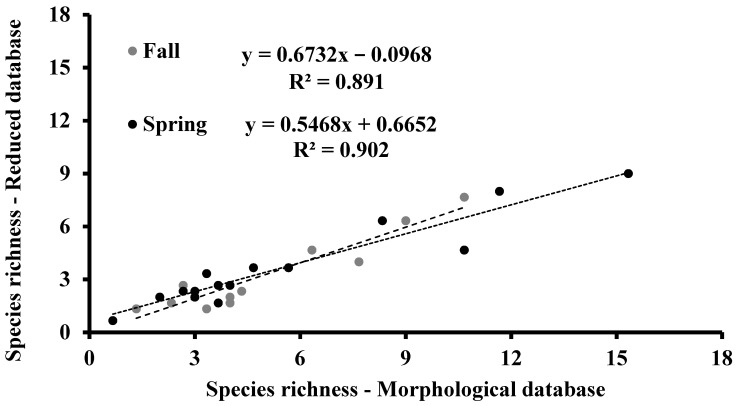
Correlation analysis between the species richness values calculated in the fall and spring sampling periods from both morphological and reduced database.

The species richness, being the simplest descriptor of the biodiversity of the ecological communities, which can also be assessed from the metabarcoding data [1], was calculated for morphological and reduced databases, as reported in Figure 2. In the fall, the percentage of species with DNA barcodes ranged from 40% in Alimini Grande (sampling site 7a) to 100% in Torre Guaceto and Punta della Contessa (sampling sites 4 and 5, respectively); 13 of the 15 percentage values obtained by comparing the morphological and the reduced databases were lower than 75%. In the spring, the percentage of species with a DNA barcode ranged from 43% in Mar Piccolo-Secondo Seno (sampling site 10) to 100% in Vasche Evaporanti Lago Salpi, Torre Guaceto, and Punta della Contessa (sampling sites 3, 4 and 5, respectively); 8 of the 15 percentage values obtained comparing the morphological and the reduced databases were lower than 75%. In each season, species-richness values based on both the morphological and the sequence data were directly and significantly correlated (*p* < 0.001; Figure 3).

### 3.3. Shannon Diversity Index

**Figure 4:**
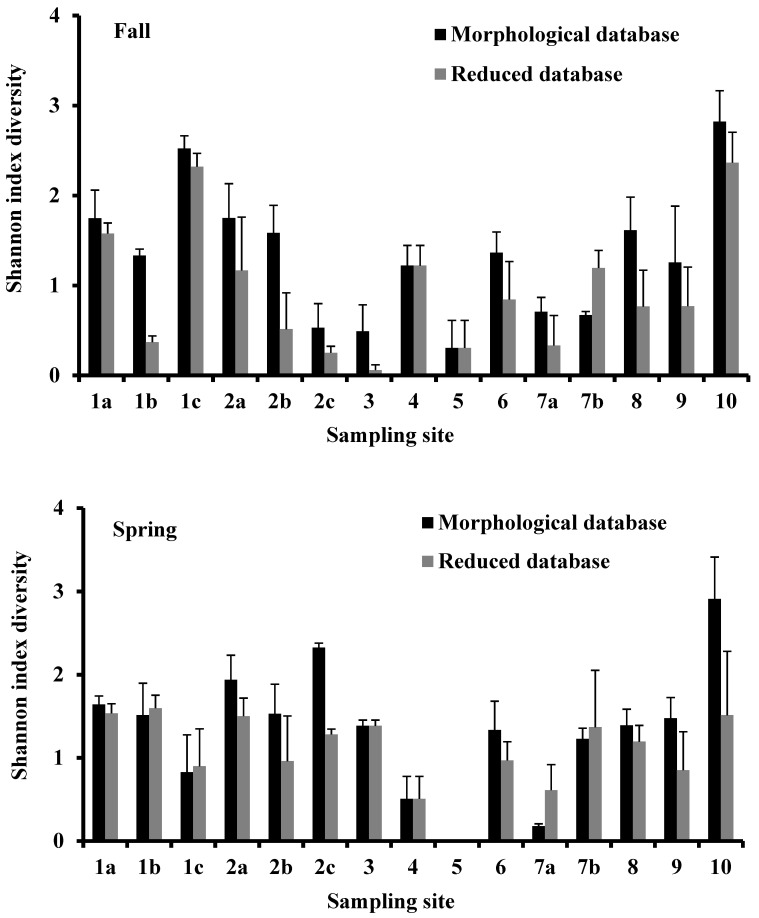
Variation of Shannon index in the sampling sites of the Apulia region transitional waters where the benthic macroinvertebrates were collected during the fall of 2010 and the spring of 2011. Vertical bars indicate the standard error.

**Figure 5:**
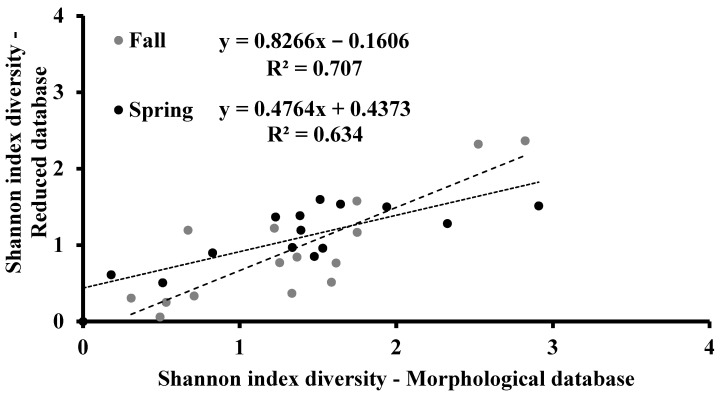
Correlation analysis between the Shannon index diversity values calculated in the fall and spring sampling periods from the morphological and reduced databases.

The Shannon diversity index takes into account the distribution of individuals among the species and was calculated for both databases. The values obtained from the morphological database were higher than those obtained from the reduced database according to the higher number of species recognized in the first one (Figure 4). Only in a few sampling sites, the index was similar or higher in the reduced database than in the morphological one. In both seasons, the Shannon diversity index values from both databases were directly and significantly correlated (*p* < 0.001; Figure 5).

### 3.4. AMBI

**Figure 6:**
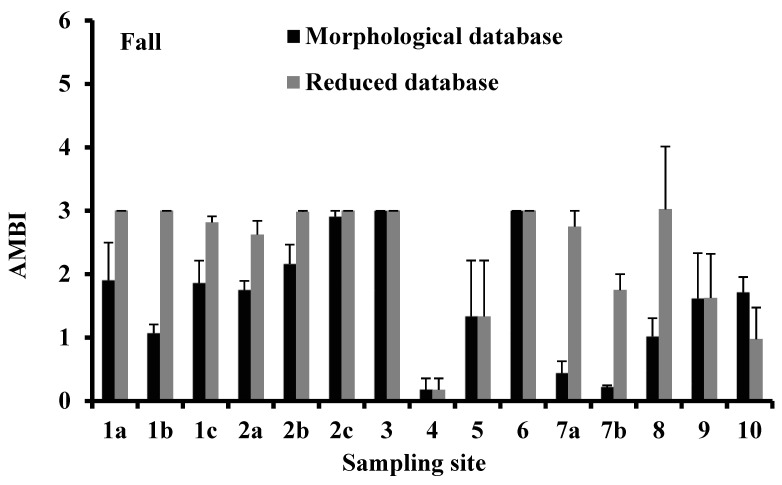

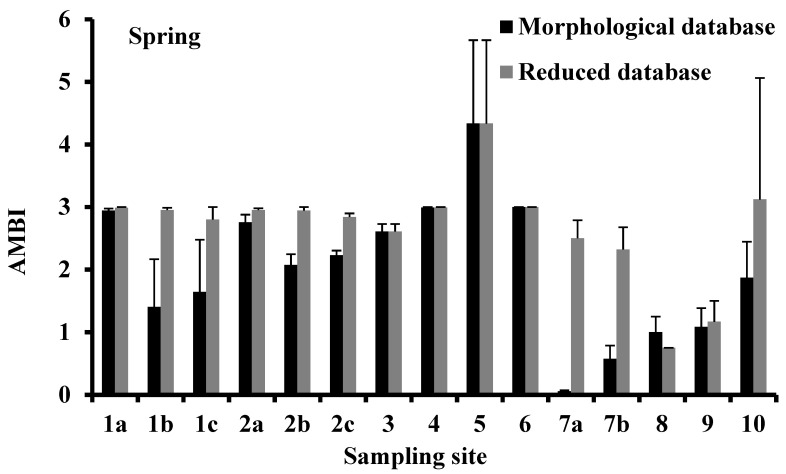
Variation of AMBI in the sampling sites of the Apulia region transitional waters where the benthic macroinvertebrates were collected during the fall of 2010 and the spring of 2011. Vertical bars indicate the standard error.

**Figure 7:**
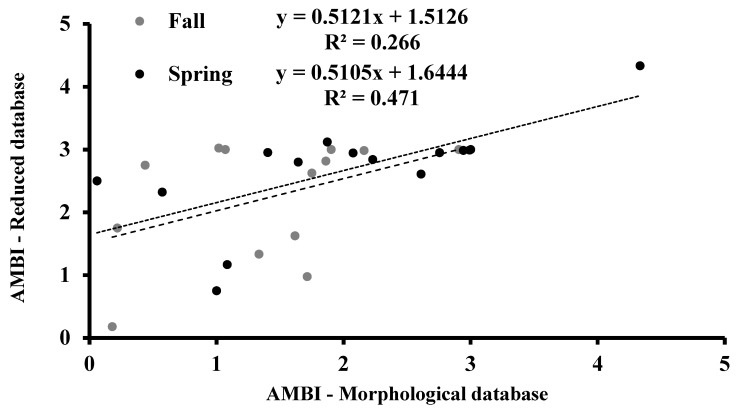
Correlation analysis between AMBI indicator values calculated in the fall and spring sampling period from the morphological and reduced databases.

According to the morphological database, the AMBI indicator was lower in the fall season than in the spring season. The lowest AMBI values in the fall were recorded in Torre Guaceto (sampling site 4), whereas in the spring, the lowest values were recorded in Alimini Grande (sampling site 7b) (Figure 6). The maximum AMBI index was recorded in the Lago di Varano (sampling site 2c) in the fall and at Punta della Contessa in the spring (sampling site 5) (Figure 6). The reduced database showed higher AMBI values than the morphological ones in both seasons (Figure 6). In each season, the AMBI values calculated from morphological and reduced databases were directly and significantly correlated (*p* < 0.001; Figure 7).

### 3.5. M-AMBI

**Figure 8:**
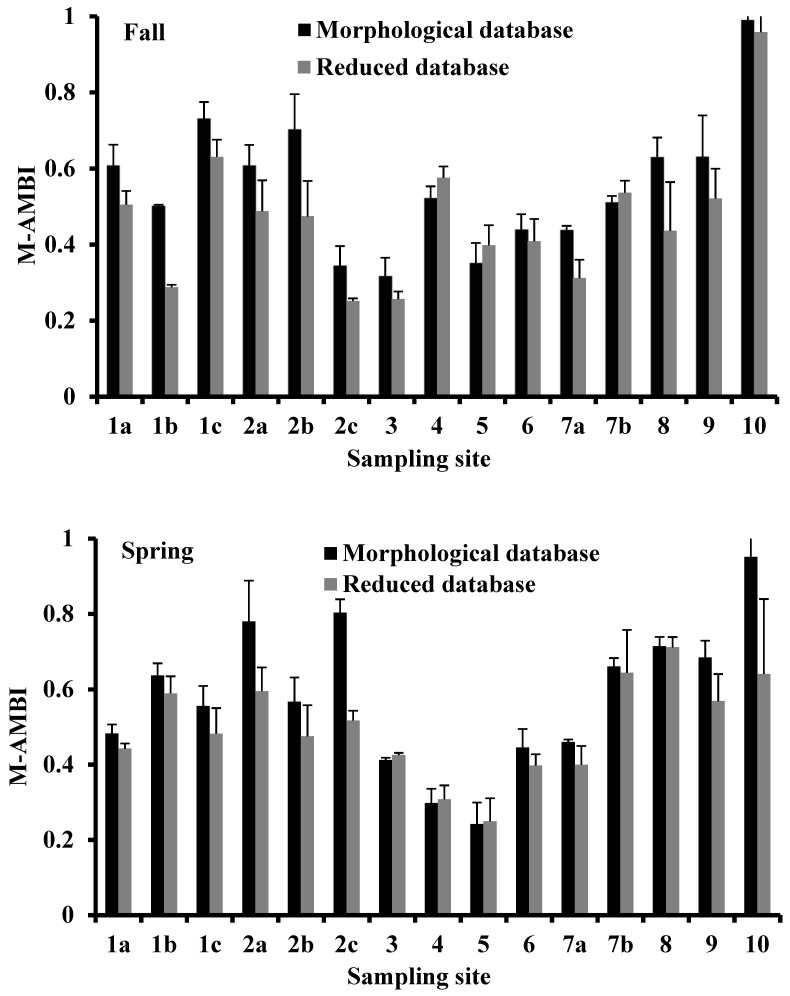
Variation of M-AMBI in the in the sampling sites of the Apulia region transitional waters where the benthic macroinvertebrates were collected during the fall of 2010 and the spring of 2011. Vertical bars indicate the standard error.

**Figure 9:**
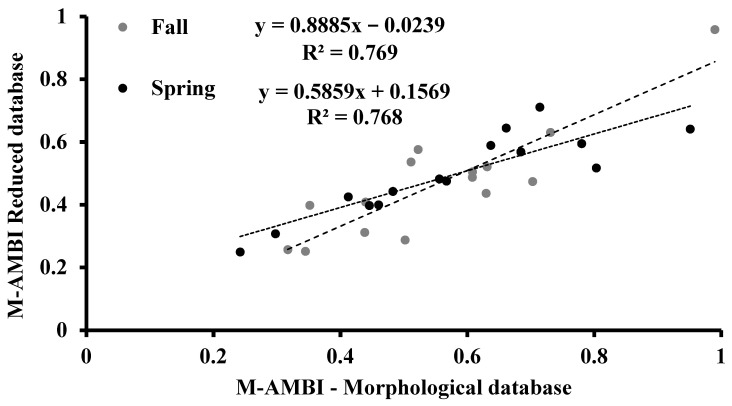
Correlation analysis between M-AMBI ecological indicator values calculated in the fall and spring sampling period from the morphological and reduced databases.

The M-AMBI indicator values were higher in the morphological database than in the reduced database in both seasons and similar between databases (Figure 8). In each season, the M-AMBI indicators from both databases were directly and significantly correlated (*p* < 0.001; Figure 9).

### 3.6. Comparison of Ecological Quality Status Assignment to the Transitional Water Ecosystems Using Morphological and DNA Barcode Databases

Using M-AMBI values, we assigned an ecological quality class to each sampling site, for each season (fall and spring), and for each reference database (morphological and DNA barcode database) (Table 1). After 30 comparisons between the morphological and barcode data, eight sites presented quality class divergences. Therefore, in 27% of the sites, the classification of the EQS obtained through the traditional approach differs from that assigned through a potential application of the DNA barcode approach. It is important to underline that the distance between the quality classifications assigned through both approaches differs on a single quality class. In addition, among the eight cases of divergence, the classification result based on the barcode database exceeds only in an instance (sampling site: 9): the one obtained from the morphological approach. This analysis and the linear relationship of the results from both approaches support the great potential of the application of DNA metabarcoding in biomonitoring programs, confirming the importance of populating the databases with barcode sequences.

### 3.7. Primers and DNA-Barcoded Region Analysis

A fundamental condition to perform a metabarcoding analysis is to identify a primer pair or primer set that anneals and amplifies the DNA of all of the species represented in the environmental sample. We checked the information of the primers used for all the listed and barcoded species in the BOLD Systems database. A primer pair is reported for 39 of the 53 barcoded species (74%), and, among these, 32 (82%) were amplified using LCO1490/HCO2198 or degenerated/modified LCO1490/HCO2198 primers (Supplementary Table S2). For the other 14 barcoded species, we verified the alignment by CLUSTALW multi-alignment with degenerated LCO1490/HCO2198 primers, and we obtained a match for eight species. This analysis outlines the importance of updating the databases with the primers used for the amplification of the DNA-barcoded region and the importance of the local/regional analysis to set the metabarcoding experiments in relation to the expected specific pool of species.

### 3.8. Interspecific Genetic Distance Analysis

**Figure 10:**
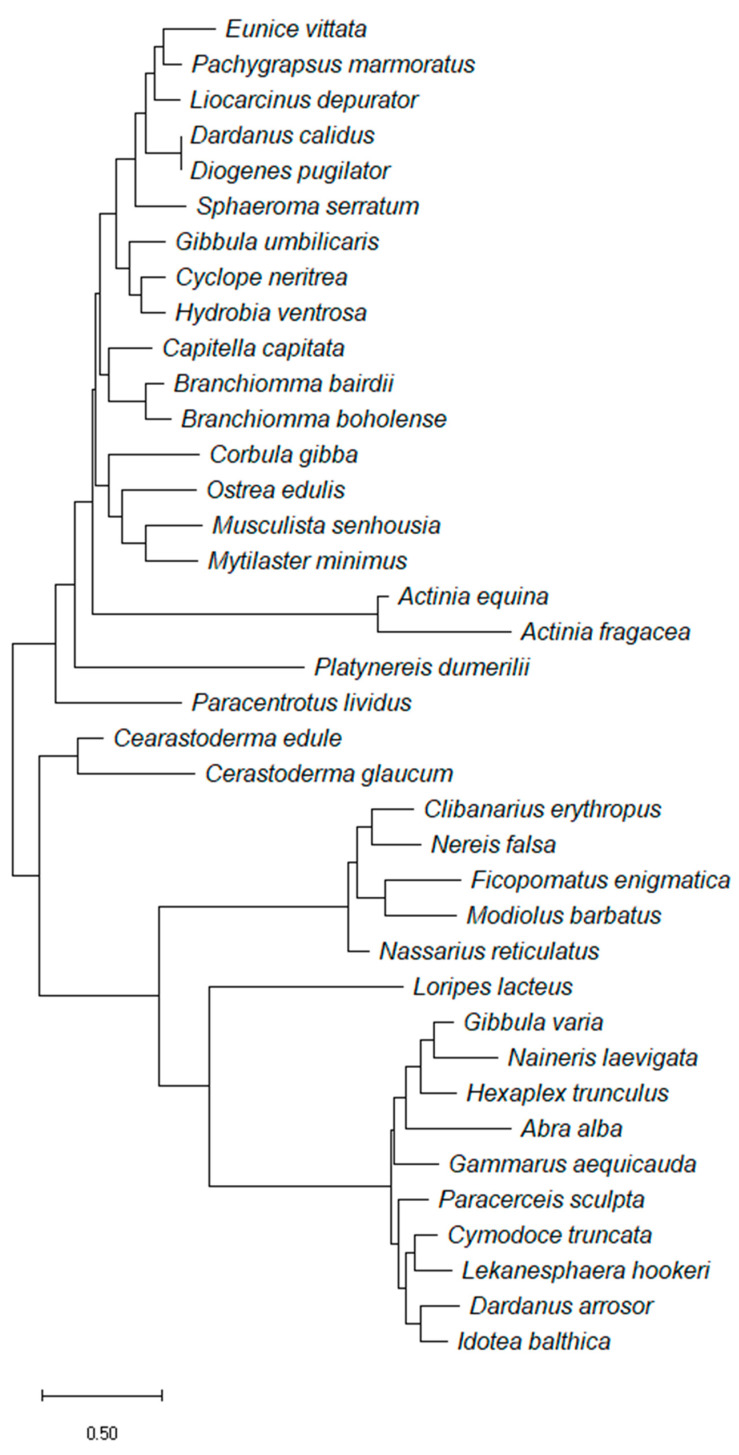
Phylogenetic analysis of consensus COI sequences available in BOLD Systems. Tree generated under the maximum likelihood criterion using the general time reversible and gamma model in RAxML. The tree is drawn to scale with branch lengths measured in number of substitutions per site.

To deepen the efficiency of the application of DNA metabarcoding, we analysed the sequences of the species present in the list to establish the interspecific genetic distances. Comparing the species delimitation results between the maximum likelihood and the neighbor-joining method (Figure 10, Tables S3 and S4), we can see that the first separates 92% of the species taken into account and exhibits Bayesian support values above 0.7 in 41%, while NJ separates 87% of the species with Bayesian support values above 0.7% in 27%. Both methods clustered together *Actinia equina* with *Actinia fragacea* and *Musculista senhousia* with *Mytilaster minimus*, but they were able to distinguish between *Dardanus arrosor* and *Dardanus calidus*, and *Gibbula umbilicaris* and *Gibbula varia*. Interestingly, *Cerastoderma edule* and *Cerastoderma glaucum* are two separate species in NJ (support value = 1.000) while they are together in ML (support value = 0.506) and, in turn, *Branchiomma bairdii* and *Branchiomma boholense* are one species in NJ (0.504) and two separate ones in ML (0.342). In addition, *Lekanesphaera hookeri* clusters with *Sphaeroma serratum* in NJ (0.281) and with *Cymodoce truncate* in ML (0.336).

## 4. Discussion

The strength of genetic identification methods to assess species biodiversity depends decisively on the completeness of the reference sequence databases. Taxa lacking barcodes in the databases cannot be identified through DNA-based approaches. Many studies show a discrepancy between molecular and morphological datasets, both in terms of species presence and abundance [45,46,47,48].

In the case study proposed in this work, the three phyla with the highest species richness (Mollusca, Arthropoda, and Annelida), covering 93% of total species in the list, present 58%, 73%, and 65% of barcoded species, respectively. The results of the analysis of DNA barcode reference libraries confirm the presence of gaps in the sampled species data coverage, comparing the Apulia Regional Environmental Protection Agency checklist with the sequences available in the BOLD Systems and GenBank, which has revealed a gap in DNA barcode sequences coverage of 42.3% [46]. Additionally, Leite et al. highlighted a lack of representative barcodes for many marine macroinvertebrate species belonging to dominant faunal groups [48].

Our results, in terms of ecological descriptors (species richness and Shannon index diversity), highlighted how the current process of identification and analysis of sampled species biodiversity through the innovative DNA barcoding approach underestimates the real biodiversity of transitional water ecosystems. Also, this trend is amplified in the transitional aquatic ecosystems where the species richness, calculated through the morphological approach, is higher [28].

In addition, 27% of the analysed transitional water ecosystems differed in the ecological quality status assigned through the potential application of both approaches. This confirms the importance of expanding barcode databases and defining useful primers sets for molecular identification at the regional level.

As proof of concept, we tried to discriminate the species present in this study solely by analysing the genetic variation of publicly available COI sequences. We took a phylogeny-based species delimitation approach by comparing two different methods: neighbor-joining and maximum likelihood. The first method finds the best tree in the dataset with a clustering algorithm, while the second considers a set of all the possible trees and selects the best based on the highest log-likelihood (lnL) tree [52]. Both methods produced a similar number of species with NJ separating 33 out of 38 species and ML separating 34 out of 38 species, but they differently designated some of the clusters. With NJ being a rapid and computationally less demanding method, it is often used to quickly separate species based on pairwise distances [53], but its accuracy decreases when applied to a large dataset, short sequences, or an unequal rate of substitution [54,55]. For these reasons, ML is increasingly used in barcoding studies [56,57,58,59,60,61] and can give a deeper resolution in terms of molecular evolution, particularly when considering a heterogeneous or previously uncharacterized dataset. This is also shown in this study where the ML approach shows higher supporting values in species delimitation. However, it is important to underline that the present analysis was obtained by building a consensus sequence from a different number of sequenced accessions found in several parts of the world. This may explain why, even though the species delimitation mostly matches our expectation, many of the reported species delimitation have low support values. In addition, molecular barcoding is often based on the amplification of a small fragment of a conserved gene, which may underestimate genetic variation and divergence time [62], and thus, incorrectly assign different species to the same cluster. We, therefore, demonstrated the importance of not only generating NGS data at a local/regional level, but also of populating reference libraries with several barcoding genes in order to fine-tune and effectively apply molecular biomonitoring.

## 5. Conclusions

DNA-based methods for assessing an ecosystem’s health are an innovative tool but require further standardization and improvement processes. This work, focused on the transitional aquatic ecosystems of the southeast Mediterranean, underlines both the importance of upgrading global DNA barcode databases and the validity of local databases for a more suitable identification of primer sets. In addition, the interspecific genetic distances analysis is relevant to verify the percentage of species potentially identified in a DNA metabarcoding study; the species with similar sequences require the analysis of other gene markers or species-specific primers for amplification of the barcoded genomic region.

## Figures and Tables

**Table 1:** Ecological quality classes of M-AMBI calculated for each lagoon/station of Apulian transitional water ecosystems, as shown in Figure 1, in the fall and spring, using both the morphological and DNA-barcode data.

Lagoon/Sampling Site	Fall		Spring	
	Morphological Database	Reduced Database	Morphological Database	Reduced Database
1a	Moderate	Poor	Poor	Poor
1b	Poor	Bad	Moderate	Moderate
1c	Moderate	Moderate	Poor	Poor
2a	Poor	Poor	Good	Good
2b	Moderate	Poor	Poor	Poor
2c	Bad	Bad	Good	Moderate
3	Bad	Bad	Bad	Bad
4	Poor	Poor	Bad	Bad
5	Bad	Bad	Bad	Bad
6	Bad	Bad	Bad	Bad
7a	Bad	Bad	Bad	Bad
7b	Poor	Poor	Moderate	Moderate
8	Moderate	Bad	Moderate	Moderate
9	Poor	Moderate	Moderate	Poor
10	High	High	Good	Moderate

## Data Availability

Not applicable.

## Supplementary Materials

The following are available online at https://www.mdpi.com/article/10.3390/biology10111092/s1, Table S1: List of taxa identified in the transitional aquatic ecosystems of Apulia (South-East Italy) for biomonitoring purposes (ARPA, 2011), Table S2: List of barcoded species and primers pair reported in the BOLD Systems database, Table S3: bPTP species delimitation based on a maximum likelihood constraint tree generated in RAxml., Table S4: bPTP species delimitation based on a Neighbor/joining constraint tree generated in MEGAX.
