# Supplementary material for: Testing the Influence of Incomplete DNA Barcode Libraries on Ecological Status Assessment of Mediterranean Transitional Waters
Source: Biology (Basel). 2021 Oct 25;10(11):1092. doi: 10.3390/biology10111092 (PMC8614736; doi:10.3390/biology10111092)
Supplement: Supplementary file 1 [file biology-10-01092-s001.zip › biology-1408386-supplementary.pdf]

biology

MDPI

Table S1. List of taxa identified in the transitional aquatic ecosystems of Apulia (South-East Italy) for biomonitoring purposes (ARPA, 2011). Transitional water ecosystems are listed as: 1 – Laguna di Lesina; 2 – Lago Varano; 3 – Vasche Evaporanti, Lago Salpi; 4 - Torre Guaceto; 5 – Punta della Contessa; 6 - Cesine; 7 - Alimini Grande; 8 - Baia di Porto Cesareo; 9 – Mar Piccolo-Primo Seno; 10 – Mar Piccolo-Secondo Seno. In each intersection between taxon and ecosystem, the number 0 or 1 corresponds to taxon absence or presence respectively.

				**Transitional water of Apulia (SE It-aly)**									
**Phylum**	**Class**	**Order**	**Taxon**	**1**	**2**	**3**	**4**	**5**	**6**	**7**	**8**	**9**	**10**
Mollusca	Bivalvia	Cardiida	*Abra alba*	0	0	0	0	0	0	0	0	0	1
Mollusca	Bivalvia	Cardiida	*Abra segmentum*	1	1	1	0	0	1	1	0	0	1
Cnidaria	Anthozoa	Actiniaria	*Actinia* sp.	0	1	0	0	0	0	0	0	0	0
Arthropoda	Malacos-	Amphipoda	*Ampelisca* sp.	0	1	0	0	0	0	0	1	0	0
Mollusca	Bivalvia	Arcida	*Anadara diluvia*	0	1	0	0	0	0	0	0	0	0
Arthropoda	Malacos-	Tanaidacea	*Apseudes latreillei*	0	0	0	0	0	0	0	1	0	0
Echinoder-	Asteroidea	Valvatida	*Asterina gibbosa*	0	0	0	0	0	0	0	0	0	1
Arthropoda	Malacos-	Decapoda	*Brachynotus sexdentatus*	0	1	0	0	0	0	0	0	0	1
Anellida	Polychaeta	Sabellida	*Branchiomma* sp.	0	0	0	0	0	0	0	0	0	1
Anellida	Polychaeta		*Capitella* sp.	0	0	0	0	0	0	0	1	0	1
Arthropoda	Malacos-	Amphipoda	*Caprella* sp.	0	0	0	0	0	0	0	0	0	1
Mollusca	Bivalvia	Cardiida	*Cerastoderma edule*	0	0	0	0	0	1	0	0	0	0
Mollusca	Bivalvia	Cardiida	*Cerastoderma glaucum*	1	1	1	0	0	1	1	0	0	0
Mollusca	Gastropoda		*Cerithium vulgatum*	0	1	0	0	0	0	0	0	0	0
Mollusca	Bivalvia	Venerida	*Chamelea gallina*	0	0	0	0	0	0	1	0	0	0
Arthropoda	Hexapoda	Diptera	*Chironomus* sp.	1	1	1	1	0	1	0	0	0	0
Anellida	Polychaeta	Terebellida	*Cirratulus* sp.	0	1	0	0	0	0	1	0	0	1
Arthropoda	Malacos-	Decapoda	*Clibanarius erythropus*	0	0	0	0	0	0	0	0	0	1
Mollusca	Bivalvia	Myida	*Corbula gibba*	0	0	0	0	0	0	0	1	0	0
Arthropoda	Malacos-	Amphipoda	*Corophium* sp.	0	1	0	0	1	0	0	0	0	0
Mollusca	Gastropoda	Neogastrop-	*Cyclope neritea*	0	1	0	0	0	0	1	0	0	0
Arthropoda	Malacos-	Isopoda	*Cymodoce truncate*	0	0	0	0	0	0	0	0	0	1
Arthropoda	Malacos-	Decapoda	*Dardanus arrosor*	0	0	0	0	0	0	0	0	0	1
Arthropoda	Malacos-	Decapoda	*Dardanus calidus*	0	0	0	0	0	0	0	1	0	1
Arthropoda	Malacos-	Decapoda	*Diogenes pugilator*	0	0	0	0	0	0	1	1	0	0
Anellida	Polychaeta		*Euclymene* spp.	0	1	0	0	0	0	0	1	0	0
Anellida	Polychaeta	Eunicida	*Eunice vittata*	0	0	0	0	0	0	0	1	1	0
Anellida	Polychaeta	Sabellida	*Ficopomatus enigmaticus*	1	0	0	0	0	0	0	0	0	0
Mollusca	Bivalvia	Pectinida	*Flexopecten flexuosus*	0	0	0	0	0	0	0	0	1	0
Arthropoda	Malacos-	Amphipoda	*Gammarus aequicauda*	1	0	1	1	0	0	0	0	0	0
Arthropoda	Malacos-	Amphipoda	*Gammarus insensibilis*	0	0	1	1	0	0	0	0	0	0
Mollusca	Bivalvia	Cardiida	*Gastrana fragilis*	0	1	0	0	0	0	1	0	1	1
Mollusca	Gastropoda	Trochida	*Gibbula umbilicaris*	0	0	0	0	0	0	0	0	1	1
Mollusca	Gastropoda	Trochida	*Gibbula varia*	0	0	0	0	0	0	0	0	1	1
Mollusca	Gastropoda		*Gibbula* sp.	0	1	0	0	0	0	0	0	0	1
Mollusca	Gastropoda	Neogastrop-	*Hadriania craticulata*	0	0	0	0	0	0	0	0	1	1
Mollusca	Gastropoda	Cephalaspi-	*Haminoea navicular*	0	1	0	0	0	0	0	0	0	1
Mollusca	Gastropoda	Neogastrop-	*Hexaplex trunculus*	0	1	0	0	0	0	0	1	0	1

Mollusca	Gastropoda	Littorinimor-	*Hydrobia ventrosa*	1	1	1	1	1	1	0	0	0	0
Anellida	Polychaeta	Sabellida	*Hydroides norvegica*	0	0	0	0	0	0	0	0	0	1
Anellida	Polychaeta	Sabellida	*Hydroides* sp.	0	1	0	0	0	0	0	0	0	0
Anellida	Polychaeta	Sabellida	*Hydroides uncinata*	0	1	0	0	0	0	0	0	0	0
Arthropoda	Malacos-	Isopoda	*Idotea balthica*	0	0	0	0	0	0	0	0	0	0
Arthropoda	Malacos-	Amphipoda	*Lekanesphaera hookeri*	1	1	0	0	0	0	0	0	0	1
Mollusca	Bivalvia	Limida	*Limaria inflate*	0	0	0	0	0	0	0	0	1	0
Arthropoda	Malacos-	Decapoda	*Liocarcinus depurator*	0	0	0	0	1	0	0	0	0	0
Mollusca	Bivalvia	Lucinida	*Loripes lacteus*	0	1	0	0	0	0	1	1	0	1
Anellida	Polychaeta	Eunicida	*Lumbrineris latreilli*	0	1	0	0	0	0	0	0	0	0
Anellida	Polychaeta		*Maldanidae indet.*	0	0	0	0	0	0	0	1	0	0
Arthropoda	Malacos-traca	Amphipoda	*Microdeutopus gryllotalpa*	0	1	0	0	0	0	0	0	0	0
Mollusca	Bivalvia	Mytilida	*Modiolus barbatus*	0	0	0	0	0	0	0	0	1	0
Mollusca	Bivalvia	Mytilida	*Musculista senhousia*	1	1	0	0	0	0	0	0	1	0
Mollusca	Bivalvia	Mytilida	*Mytilaster minimus*	1	1	0	0	0	0	1	0	0	0
Anellida	Polychaeta		*Naineris laevigata*	0	0	0	0	0	0	0	0	0	1
Mollusca	Gastropoda	Neogastrop-	*Nassarius corniculum*	0	0	0	0	0	0	0	0	1	0
Mollusca	Gastropoda	Neogastrop-	*Nassarius cuvieri*	0	0	0	0	0	0	0	0	0	1
Mollusca	Gastropoda	Neogastrop-	*Nassarius reticulatus*	0	1	0	0	0	0	1	0	1	1
Anellida	Polychaeta	Phyllodocida	*Neanthes arenaceodentata*	0	0	0	0	0	0	0	0	0	1
Anellida	Polychaeta	Phyllodocida	*Neanthes succinea*	1	1	0	0	0	0	0	0	0	0
Anellida	Polychaeta	Phyllodocida	*Nephtys* sp.	0	1	0	0	0	0	0	0	0	0
Anellida	Polychaeta	Phyllodocida	*Nereis diversicolor*	1	1	0	1	0	0	0	0	0	0
Anellida	Polychaeta	Phyllodocida	*Nereis falsa*	1	1	0	0	0	0	0	0	0	0
Anellida	Polychaeta	Phyllodocida	*Nereis* sp.	1	1	0	0	1	0	1	0	0	1
Mollusca	Gastropoda		*Odostomia* sp.	0	1	0	0	0	0	0	0	0	0
Mollusca	Bivalvia	Ostreida	*Ostrea edulis*	0	0	0	0	0	0	0	0	0	1
Arthropoda	Malacos-traca	Decapoda	*Pachygrapsus marmoratus*	0	1	0	0	0	0	0	0	0	1
Arthropoda	Malacos-	Decapoda	*Paguristes eremita*	0	0	0	0	0	0	0	0	0	1
Echinoder-	Echinoidea	Camarodonta	*Paracentrotus lividus*	0	0	0	0	0	0	0	0	1	0
Arthropoda	Malacos-	Isopoda	*Paracerceis sculpta*	0	0	0	0	0	0	0	0	0	1
Anellida	Polychaeta	Phyllodocida	*Perinereis macropus*	0	1	0	0	0	0	0	0	0	0
Sipuncula	Sipuncu-		*Phascolion strombus strombus*	0	0	0	0	0	0	0	1	0	0
Arthropoda	Malacos-	Decapoda	*Pisidia longimana*	0	0	0	0	0	0	0	0	0	1
Anellida	Polychaeta	Phyllodocida	*Platynereis dumerilii*	0	1	0	0	0	0	0	0	0	0
Anellida	Polychaeta		*Polychaeta indet.*	0	1	0	0	0	0	1	0	0	0
Mollusca	Gastropoda	Neogastrop-	*Raphitoma* sp.	0	0	0	0	0	0	0	1	0	0
Anellida	Polychaeta	Sabellida	*Serpula vermicularis*	1	0	0	0	0	0	0	0	0	0
Arthropoda	Malacos-	Isopoda	*Sphaeroma serratum*	0	0	0	0	0	1	0	0	0	1
Anellida	Polychaeta	Phyllodocida	*Syllis* sp.	0	0	0	0	0	0	0	0	0	1
Mollusca	Bivalvia	Venerida	*Tapes decussatus*	0	1	0	0	0	0	1	0	0	1
Mollusca	Bivalvia	Venerida	*Tapes philippinarum*	0	1	0	0	0	0	1	0	0	1
Mollusca	Bivalvia	Cardiida	*Tellina planata*	0	0	0	0	0	0	1	0	0	0
Mollusca	Bivalvia	Cardiida	*Tellina* sp.	0	0	0	0	0	0	0	1	0	0

Table S2. List of barcoded species and primers pair reported in the BOLD systems database

SPECIES NAME	PRIMERS
Abra alba	dgHCO-2198/dgLCO-1490
Ampelisca sp	CrustDF1 / CrustDR1
Branchiomma sp.	jgLCO1490 / jgHCO2198
Capitella sp.	C_VF1LFt1 / C_VR1LRt1
Caprella sp.	LCO1490_t1 / HCO2198_t1
Cerastoderma edule	LCO1490 / HCO2198
Chironomus sp.	LCO1490 / HCO2198
Cirratulus sp.	jgLCO1490 / jgHCO2198
Clibanarius erythropus	LCO1490_t1 / HCO2198_t1
Corbula gibba	COIF-ALT / COIR-ALT
Corophium sp.	jgLCO1490 / jgHCO2198
Cymodoce truncata	LCO1490_t1 / HCO2198_t1
Dardanus arrosor	LCO1490 / HCO2198
Dardanus calidus	CrustF1 / HCO2198
Diogenes pugilator	LCO1490 / HCO2198
Euclymene spp.	LoboF1 / LoboR1
Gibbula umbilicaris	LCO1490 / HCO2198
Gibbula varia	LCO1490 / HCO2198
Hexaplex trunculus	LCO1490 / HCO2198
Idotea balthica	LCO1490 / HCO2198
Liocarcinus depurator	LCO1490 / HCO2198
Liocarcinus depurator	LCO1490 / HCO2198
Lumbrineris latreilli	LCO1490 / HCO2198
Maldanidae indet.	LCO1490 / polyHCO
Modiolus barbatus	jgLCO1490 / jgHCO2198
Naineris laevigata	dgLCO-1490 / dgHCO-2198
Nephtys sp.	LCO1490_t1 / HCO2198_t1
Nereis falsa	C_VF1LFt1 / C_VR1LRt1 and LoboF1 / LoboR1
Nereis sp.	jgLCO1490 / jgHCO2198
Odostomia sp.	LCO1490_t1 / HCO2198_t1 and jgLCO1490 / jgHCO2198
Ostrea edulis	LoboF1 / LoboR1
Pachygrapsus marmoratus	LCO1490 / HCO2198
Paracerceis sculpta	jgLCO1490 / jgHCO2198
Raphitoma sp.	polyLCO / polyHCO
Serpula vermicularis	LCO1490 / HCO2198
Sphaeroma serratum	jgLCO1490 / jgHCO2198
Tellina sp.	veneroidLCO / HCO2198
Nassarius reticulatus	LCO1490 / HCO2198

Table S3. bPTP species delimitation based on a maximum likelihood constraint tree generated in RAxml. Number in brackets represent the number of sequences retrieved from BOLD to build the consensus sequence of each species.

Species no	Species name (Number of sequences)	Support value
1	*Abra alba* (7)	0.574
2	*Actinia equina* (6), *Actinia fragacea* (1)	0.504
3	*Eunice vittata* (1)	1
4	*Branchiomma bairdii* (75)	0.342

5	*Branchiomma boholense* (48)	0.342
6	*Capitella capitata* (32)	0.673
7	*Cerastoderma edule* (125), *Cerastoderma glaucum* (226)	0.509
8	*Clibanarius erythropus* (12)	0.351
9	*Corbula gibba* (7)	0.757
10	*Cyclope neritrea* (42)	0.344
11	*Cymodoce truncata* (6), *Lekanesphaera hookeri* (2)	0.336
12	*Dardanus arrosor* (18)	0.354
13	*Dardanus calidus* (5)	0.504
14	*Diogenes pugilator* (12)	0.504
15	*Ficopomatus enigmaticus* (9)	1
16	*Gammarus aequicauda* (6)	0.755
17	*Nereis falsa* (1)	0.351
18	*Gibbula umbilicaris* (25)	0.657
19	*Gibbula varia* (30)	0.183
20	*Hexaplex trunculus* (128)	0.373
21	*Hydrobia ventrosa* (46)	0.344
22	*Idotea balthica* (17)	0.354
23	*Liocarcinus depurator* (107)	1
24	*Loripes lacteus* (12)	0.956
25	*Modiolus barbatus* (60)	0.324
26	*Musculista senhousia* (53), *Mytilaster minimus* (14)	0.248
27	*Naineris laevigata* (11)	0.183
28	*Nassarius reticulatus* (55)	0.834
29	*Ostrea edulis* (29)	0.489
30	*Pachygrapsus marmoratus* (58)	1
31	*Paracentrotus lividus* (258)	0.99
32	*Paracerceis sculpta* (19)	0.805
33	*Platynereis dumerilii* (66)	0.993
34	*Sphaeroma serratum* (3)	1

Table S4. bPTP species delimitation based on a Neighbor/joining constraint tree generated in MEGAX. Number in brackets represent the number of sequences retrieved from BOLD to build the consensus sequence of each species.

**Specie no**	**Species name (Number of sequences)**	**Support value**
1	*Abra alba* (7)	1.000
2	*Actinia equina* (6), *Actinia fragacea* (1)	0.513
3	*Eunice vittata* (1)	0.734
4	*Branchiomma bairdii* (75) , *Branchiomma boholense* (48)	0.504
5	*Capitella capitata* (32)	1.000
6	*Cerastoderma edule* (125)	1.000
7	*Cerastoderma glaucum* (226)	1.000
8	*Clibanarius erythropus* (12) , *Gammarus aequicauda* (6)	0.506

9	*Corbula gibba* (7)	1.000
10	*Cyclope neritrea* (42)	0.383
11	*Cymodoce truncata* (6)	0.553
12	*Dardanus arrosor* (18)	0.568
13	*Dardanus calidus* (5)	0.302
14	*Diogenes pugilator* (12)	0.302
15	*Ficopomatus enigmatica* (9)	0.743
16	*Nereis falsa* (1)	0.262
17	*Gibbula umbilicaris* (25)	0.304
18	*Gibbula varia* (30)	0.304
19	*Hexaplex trunculus* (128)	0.367
20	*Hydrobia ventrosa* (46)	0.367
21	*Idotea balthica* (17)	0.405
22	*Lekanesphaera hookeri* (2), *Sphaeroma serratum* (3)	0.281
23	*Liocarcinus depurator* (107)	0.424
24	*Loripes lacteus* (12)	1.000
25	*Modiolus barbatus* (60)	0.509
26	*Musculista senhousia* (53), *Mytilaster minimus* (14)	0.254
27	*Naineris laevigata* (11)	0.494
28	*Nassarius reticulatus* (55)	0.383
29	*Ostrea edulis* (29)	1.000
30	*Pachygrapsus marmoratus* (58)	0.424
31	*Paracentrotus lividus* (258)	0.597
32	*Paracerceis sculpta* (19)	0.405
33	*Platynereis dumerilii* (66)	0.262
